# Role of bestatin as a treatment for periodontitis

**DOI:** 10.3389/fdmed.2025.1571989

**Published:** 2025-05-13

**Authors:** Sudhir Rama Varma, Bader Mohamed Moustafa Elagha, Jayaraj K. Narayanan, Asok Mathew

**Affiliations:** ^1^ Department of Clinical Sciences, Ajman University, Ajman, United Arab Emirates; ^2^ Center for Medical and Bio-Allied Health Sciences Research, Ajman University, Ajman, United Arab Emirates; ^3^ Department of Basic Sciences, Ajman University, Ajman, United Arab Emirates

**Keywords:** bestatin, antimicrobial therapy, *Porphyromonas gingivalis*, aminopeptidase inhibitor, biofilm, periodontitis

## Abstract

Periodontitis is a chronic inflammatory disease impacting the supporting structures of teeth, with significant global pervasiveness and systemic health implications. Current treatments, such as scaling and root planning (SRP) and adjunctive antibiotics, face challenges including antibiotic resistance, infection recurrence, and incomplete tissue regeneration. Bestatin, a dipeptide aminopeptidase inhibitor, has shown potential as a novel therapeutic agent due to its targeted antimicrobial effects against *Porphyromonas gingivalis (P. gingivalis),* biofilm modulation, and anti-inflammatory properties. *in vitro* studies revealed bestatin's selective bacteriostatic effects against P. gingivalis, inhibiting bacterial growth and biofilm development without affecting commensal bacteria. *in vivo* studies demonstrated that bestatin modulated inflammatory responses and prevented necrotic abscess formation in guinea pig models, suggesting its potential to suppress *P. gingivalis* growth through alternative pathways. However, no clinical trials were identified, highlighting a significant gap in the translation of preclinical findings into human periodontal therapy. The current review highlights Bestatin as a promising therapeutic representative for periodontitis, where it is involved in inhibiting modulating biofilms, reducing tissue destruction, and *P. gingivalis*, in preclinical studies. Compared to traditional therapies, bestatin provides unique advantages, non-cytotoxicity, including specificity, and dual action against microbial dysbiosis along with biofilm-associated resistance.

## 1 Introduction

### 1.1 Overview of periodontitis, prevalence and systemic impact

Periodontitis is a complex inflammatory condition of the structures adjacent to teeth, involving the periodontal ligament, gingiva, and alveolar bone (1, 2). This chronic condition, if left untreated, turns to mobility and eventual loss of tooth, critically impacting the nature of life (3). The universal pervasiveness of periodontitis is consequential, with studies showing its incidence to influence up to 50% of the grown population worldwide, differing in severity (4, 5). However, its impact is non-restricted to oral health, systemic involvement includes cardiovascular disease, respiratory diseases, adverse pregnancy outcomes, and diabetes mellitus (6, 7).

### 1.2 Current treatment approaches for periodontitis and its limitations

Current treatments initially involve mechanical debridement, like scaling along with root planning (SRP), anticipated to remove subgingival calculus and pathogenic bacterial biofilms. Adjunctive therapies, and systemically or locally delivered antiseptics and antibiotics, have been reported to increase the effectiveness of SRP (8). However, concerns like the rise in antibiotic resistance, incomplete regeneration of damaged periodontal tissues, and the recurrence of infection highlight significant resistance to current approaches (9). These challenges highlight the urgency of identifying adjunctive treatments that curb microbial proliferation as well as restore the integrity of periodontal tissue.

### 1.3 Bestatin: a potential adjunctive therapy

**Figure 1:**
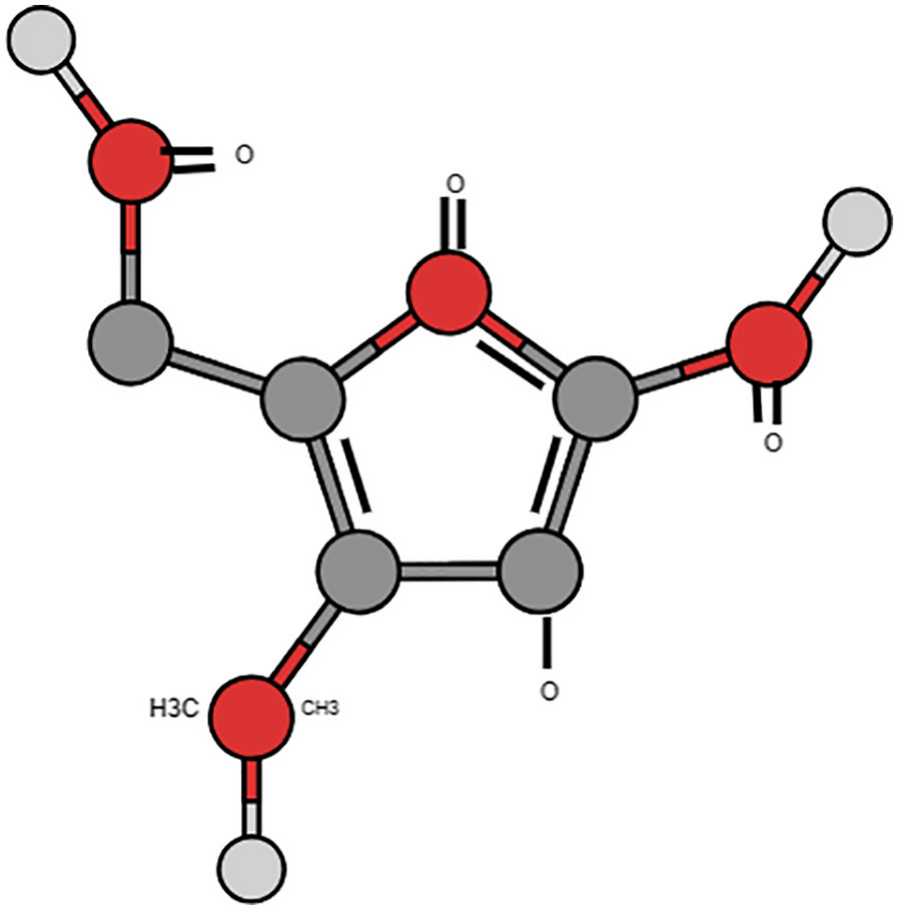
Structure of bestatin molecule.

The molecular name for bestatin is [(2S,3R)-3-amino-2-hydroxy-4-phenylbutanoyl]. The small dipeptide L-leucine is derived from Streptomyces olivoreticuli (10) (Figure 1). Its natural activity stems from its part as an aminopeptidase inhibitor. Aminopeptidases, a kind of proteolytic enzymes, are significant in peptide metabolism and implicated in immune modulation including inflammatory responses (11). Its potential role in periodontal conditions seems promising as few studies have shown its effect primarily against the keystone pathogen *P. gingivalis* part of the red complex group of bacteria (12, 13).

### 1.4 Anti-microbial effects against *P. gingivalis*

Preclinical studies prove that bestatin demonstrates the broad-spectrum antimicrobial characteristics, notably against important periodontal pathogens like the red complex and the green complex group of bacteria, but noteworthy from literature is its role against *P. gingivalis* (*Porphyromonas gingivalis,* which is part of the red complex group of bacteria (12). Studies have evaluated its role and validated its role in the etiology of periodontitis, providing biofilm maturation, tissue destruction, and immune evasion through its collection of virulence factors and gingipains (12, 13). Bestatin's focused inhibitory gesture on *P. gingivalis* growth, like demonstrated in *in vitro* studies, is of main interest for periodontal therapy (13). The immunomodulatory qualities of bestatin suggest that it may lessen periodontal inflammation and act as a marker to lessen the severity of the disease (12).

### 1.5 *in vivo* studies on mitigating inflammatory activities

Bestatin, is a dipeptide evolved from *Streptomyces olivoreticuli*, has gained attention for its ability to inhibit aminopeptidases and play an important part in modulating inflammation along with immune responses (14, 15). Current *in vitro* studies focus bestatin's selective antimicrobial potency against *P. gingivalis*, a mainstay pathogen in periodontitis. Kaminska, Benedyk-Machaczka (12) reported that bestatin essentially inhibited the proteolytic act of *P. gingivalis*, thus intruding with its virulence. Labbé, Grenier (16) showed that bestatin stifled bacterial growth along with biofilm formation, proposing its potential to distort the pathogenic microbial environment features of periodontitis. Over its antimicrobial effectiveness, bestatin's anti-inflammatory attributes have been demonstrated in preclinical models, modulated immune responses, and decreased inflammatory cytokine levels (17). These properties align with the therapeutic requirements of periodontitis and involve both microbial suppressions along with inflammation control to halt disease progression as well as promote periodontal regeneration.

The increasing body of evidence underlines the promise of bestatin like an adjunctive therapy competent in identifying the limitations of current treatments. However, the translation of these findings into clinical practice remains underexplored, with limited studies investigating its effects on periodontal tissues *in vivo* or its clinical efficacy in human populations. This difference in the literature requires a comprehensive review to consolidate existing knowledge and identify research priorities. Thus, the current review aims to map bestatin's role in periodontitis treatment, focusing on its antimicrobial, anti-inflammatory, and tissue-preserving effects. By synthesizing findings from *in vitro*, *in vivo*, and clinical studies, this review seeks to provide a foundation for further research along with inform the potential integration of bestatin into periodontal therapy protocols.

### 1.6 Research questions

1. What is the *in vitro* evidence for bestatin's efficacy against *P. gingivalis*?
2. What are the *in vivo* effects of bestatin on periodontal inflammation and tissue destruction?
3. What is the clinical evidence for bestatin's efficacy in treating periodontitis in humans?

The current review aims to collate and evaluate the available evidence regarding bestatin's potential as a treatment for periodontitis. The findings were examined through three main research questions, focusing on bestatin's efficacy against *P. gingivalis in vitro*, its effects on periodontal inflammation and tissue destruction *in vivo*, and its clinical relevance in managing periodontitis.

### 1.7 Role of bestatin in inhibiting dysbiosis

The *in vitro* studies in this review continuously focuses on bestatin's remarkable inhibitory impact on *P. gingivalis*, a cornerstone pathogen in the etiology of periodontitis. Grenier (1992) (18) reported that bestatin decreased the growth of *P. gingivalis* more successfully than other protease inhibitors, like iodoacetamide and leupeptin (19, 20). Mainly, bestatin's action was particular for *P. gingivalis* without influencing any other oral bacterial species (21). This particularly proposes that bestatin could focused pathogenic bacteria during protecting commensal flora, an aspect that coordinates with the requirement for accuracy in periodontal therapies (22). Earlier research has highlighted the importance of restoring the oral microbiome to managing systemic and oral health, making bestatin's particular action an important advantage (23). Grenier along with Michaud (1994) (19) further polished the concept of bestatin's mechanism, observing that its impact were bacteriostatic instead of bactericidal. Unlike conventional antibiotics, that frequently kill bacteria completely, bestatin prevents bacterial proliferation. This difference is critical due to bactericidal agents can instigate resistance mechanisms, while bacteriostatic agents such as bestatin may decrease specific pressure on microbial flora (24). Notably, current study found that bestatin's inhibitory impacts were not related to its activity like an aminopeptidase inhibitor, showing that its mechanism of action might include disrupting other metabolic pathways essential for *P. gingivalis* growth (21).

**Figure 2:**
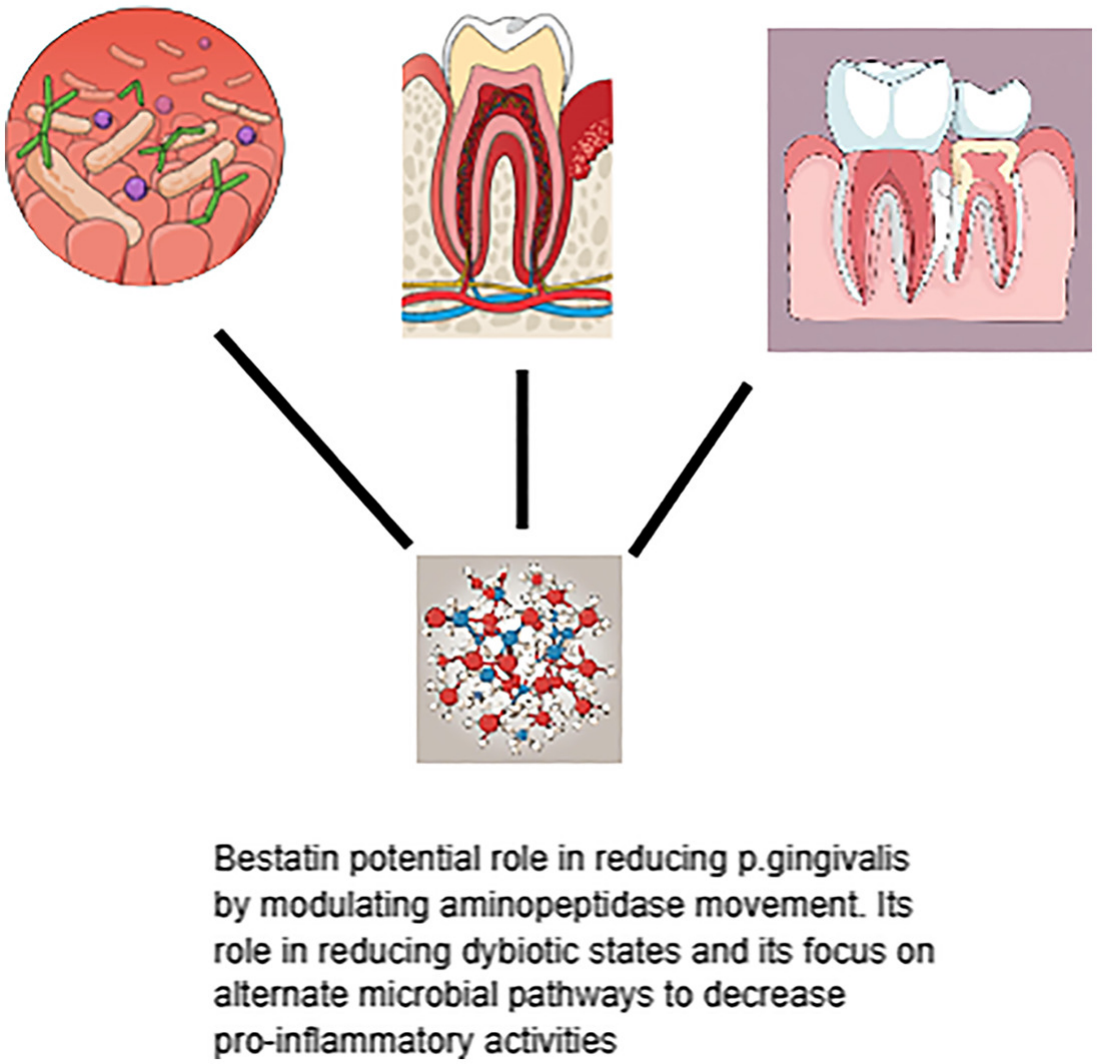
Role of bestatin in periodontal treatment.

Kaminska et al. (2023) (12) included a vital dimension to this testimony by analyzing bestatin's effects on biofilms, the primary microbial population in periodontitis. Their research reported that bestatin efficiently manages biofilm development along with species composition in mono- as well as multispecies models. As biofilms confer crucial resistance to traditional antimicrobial agents (25), bestatin's biofilm-modulating attributes represent an advancement. These outcomes recommend that bestatin could enhance mechanical debridement by decreasing biofilm-associated resistance, a significant hurdle in periodontal therapy. Fascinatingly, Labbé et al. (2001) (16) reported that bestatin disturbs *P. gingivalis* growth by restricting the intracellular intake of peptides and amino acids, vital for nitrogen metabolism and bacterial energy. This mainly focusing on metabolic pathways further identifies bestatin from traditional antibiotics, and often has broader along with less targeted effects (26). However, Labbé et al. ensured that bestatin is non-cytotoxic to host cells, assisting its safety for specific applications in periodontal pockets (16) (Figure 2).

The *in vitro* evidence from this review coordinates with previous studies emphasizing the requirement for specific antimicrobial therapies in periodontitis. For example, researchers on alternative therapies such as bacteriophages and antimicrobial peptides have similarly underlined the significance of specificity in decreasing pathogenic bacterial communities while sparing advantageous microbes (27). Contrasted to these evolving therapies, bestatin's dual part modulates biofilm positions and inhibits *P. gingivalis* as a promising agent.

### 1.8 Role of bestatin as an anti-cancer agent

Recent advances related to Bestatin are reviewed. Bestatin had been recognized as an immunomodulator for anti-cancer drugs, but recently Bestatin has been shown to induce an *in vitro* anti-tumor effect on bladder cell carcinoma, breast cancer and leukemic cells by inducing apoptosis. Caspase-3 activation is a key event in apoptosis induced by Bestatin (29). Leukotriene A4 hydrolase (LTA4H) plays an important role in cancer development and its presence in upregulating key cancer pathophysiology in certain type of cancers such as thyroid cancer, esophageal and lung cancer have been studied (28). The potential role of bestatin in mitigating upregulation of LTA4H and its receptors and thereby reducing tumor growth have been studied (29, 30). The potential possibility of use of bestatin to mitigate dysbiotic pathways pertaining to periodontitis cannot be ignored.

### 1.9 Alternate pathway by bestatin in disrupting biofilm nutrient acquisition

The *in vivo* research reviewed gives preliminary proof of bestatin's capability to mitigate periodontal inflammation along with tissue destruction, important characteristics of periodontitis. Labbé et al. (2001) (16) reported that bestatin injections averted necrotic abscess formation in a guinea pig model as opposed to *P. gingivalis*. This result is significant cause it underlines bestatin's potential to distort the pathogen's capability to induce devastating inflammatory responses, a trademark of periodontitis progression. The capability to manage inflammation while controlling host tissue integrity differentiates bestatin from traditional therapies that consistently focuses particularly on bacterial eradication. Grenier et al. (2001) (20) analysed bestatin's part in modulating aminopeptidase movement in *P. gingivalis*. They marked that when bestatin did not inhibit the initial aminopeptidase activities (dipeptidyl aminopeptidase IV, and arginine aminopeptidase), it uniquely overcame *P. gingivalis* growth. This result suggests that bestatin can act through replacement mechanisms that do not involve immediate enzyme inhibition, like biofilm formation or disrupting nutrient acquisition. This mechanism makes it as unique to aminopeptidase inhibitors along with broad-spectrum antibiotics and often affects both pathogenic as well as commensal bacteria indiscriminately.

### 1.10 Potential of bestatin as local delivery agent

In Contrast to host-modulating agents like sub-antimicrobial-dose doxycycline (SDD), and targeting matrix metalloproteinases related to decrease tissue destruction (27), bestatin proposes complementary strategies by directly focusing microbial metabolic pathways. When SDD has reported efficacy in stabilizing periodontal tissues, its systemic management raises issues about side effects along with long-term safety (31). Bestatin's potential for regional application could reduce these risks, proposing a safer alternative for controlling periodontal inflammation. Moreover, it is important to mark the limited area of *in vivo* research on bestatin. The absence of data on its prolonged effects on tissue regeneration and inflammation represents a considerable gap. Previous studies on therapies such as minocycline microspheres have shown sustained decreases in periodontal inflammation along with pocket depth for long periods (32).

## 2 Future directions

Future study directions for bestatin as a therapeutic agent in periodontitis encompass multiple vital areas. Firstly, meticulous clinical trials are important to evaluate bestatin's efficacy in updating key clinical results, including probing depth depletion, bleeding on probing, along with attachment level gain. This research will provide proof of its practical appeal in periodontal therapy. Secondly, exploring the synergistic effects of bestatin in tandem with existing treatments, like host-modulating agents or scaling along with root planing, could offer intuition into its potential like an adjunctive therapy. Thirdly, longitudinal studies are required to evaluate the influence of bestatin on periodontal stability including tissue regeneration over prolonged periods, addressing a remarkable gap in the current study. Additionally, mechanistic research should investigate bestatin's effects on biofilms including host immune responses, increasing our understanding of its therapeutic potential. However finally, upgradation in drug delivery systems, like nanoparticles and hydrogels, could enable sustained and localized release of bestatin in periodontal pockets, enhancing its efficacy along with patient compliance and offer an effective and viable option in current periodontal therapy.

Kaminska et al. (2023) (12) highlights the requirement for rigorous clinical trials to point out bestatin's therapeutic potential as well as establish its safety along with efficacy in humans. Earlier clinical studies on adjunctive treatment for periodontitis, like antiseptics and antibiotics, have reported variable success (33, 34, 35). In particular, amoxicillin and metronidazole have been reported to reduce pocket depths and improve clinical attachment levels when used as adjuncts to scaling along with root planning (36). Moreover, these therapies are related to systemic side effects including the potential for antibiotic resistance, focusing on the requirement for alternatives such as bestatin. Dissimilar to systemic antibiotics, bestatin's specific application could reduce systemic exposure along with associated risks, referring to it as a safer choice for extended management.

However, antiseptics such as chlorhexidine, are effective in minimizing bacterial load and are related to adverse effects like altered taste and staining (37). Bestatin's specificity and non-cytotoxic nature to *P. gingivalis* recommend that it could provide akin antimicrobial welfare without these downsides. Moreover, clinical trials are vital to confirm these advantages along with determining bestatin's efficacy in upgrading clinical bounds like probing depth, clinical attachment level including bleeding on probing.

When conventional therapies were compared, bestatin exhibits multiple unique advantages. Antibiotics such as amoxicillin and metronidazole, while effective and hostile to periodontal pathogens, pose a risk of systemic adverse effects, and the development of resistance (38). Bestatin's focused action against *P. gingivalis* along with its bacteriostatic mechanism may minimize these risks, presenting a more sustainable perspective to periodontal therapy. Host-modulating therapies, like sub-antimicrobial-dose doxycycline, have been studied in detail for their capability to inhibit matrix metalloproteinases as well as reduce inflammation (27). Stint effective, these therapies need systemic administration and carry the potential for side effects. Bestatin's area-specific application could give a similar anti-inflammatory advantage without systemic exposure, ranging from the growing trend to precision medicine in periodontal treatment. Biofilm resistance is an additional censorious challenge in periodontitis treatment. Traditional antimicrobials normally fight to penetrate biofilms, minimizing their effectiveness (39). Bestatin's reported ability to regulate biofilm composition along with inhibiting *P. gingivalis* growth in biofilm model locations is a valuable inclusion to existing therapeutic choices. This finding ranges with recent studies emphasizing the significance of disrupting biofilm building design to enhance treatment results (40).

### 2.1 Limitations and implications for clinical practice

The findings from the current review proposes that bestatin has notable potential as an adjunctive treatment for periodontitis. Its antimicrobial action, non-cytotoxic nature, and biofilm-modulating properties make it a significant alternative to traditional antiseptics and antibiotics. However, the insufficiency of clinical data available is an essential barrier to its integration into quality periodontal care. Regardless of the promising preclinical results, no clinical trials have been run to estimate bestatin's effectiveness in human recipients with periodontitis. Bestatin is currently marketed by the name Ubenimex (Nippon Kayaku; Japan). It is also marketed as Bestatin™ in Japan. It does not have FDA clearance to be marketed and used in the Unites states of America and Europe. Due to the lack of the drug availability, its potential role in treating inflammatory conditions from a clinical point of view remains non-evident. This lack of clinical data is a prime limitation, as it limits the conveying of laboratory outcomes in conventional periodontal treatment procedures. Future clinical trials should rely on accessing bestatin's efficacy in the merger with scaling along with root planing, the benchmark for periodontal therapy. Research should also investigate ideal delivery systems, like localized applications via gels, nanoparticles, or microspheres to maximize their therapeutic effects at the time of minimizing systemic exposure.

## 3 Conclusion

Bestatin has appeared as a potential therapeutic agent for periodontitis, with demonstrated role of impeding *P. gingivalis* in microbial colonies, and furthermore reducing tissue destruction, and modulating biofilms in preclinical research. Compared to traditional therapies, bestatin offers distinctive advantages, including non-cytotoxicity, specificity, and dual action as opposed to microbial dysbiosis along with biofilm-associated resistance. The scarcity of studies currently limits clinical data, highlighting the necessity for further research to ascertain its significance in periodontal treatment. Further *in vitro* studies are necessary to comprehensively assess its potential in mitigating dysbiotic activities associated with periodontal inflammation. By addressing these gaps, bestatin could potentially transform the management of periodontitis, providing a targeted and focused approach.
